# Unicode-8 based linguistics data set of annotated Sindhi text

**DOI:** 10.1016/j.dib.2018.05.062

**Published:** 2018-05-22

**Authors:** Mazhar Ali Dootio, Asim Imdad Wagan

**Affiliations:** a Shaheed Zulifqar Ali Bhutto Institute of Science & Technology (SZABIST), Karachi, Sindh, Pakistan; b Benazir Bhutto Shaheed University Lyari, Karachi, Sindh, Pakistan; c Mohammad Ali Jinnah University, Karachi, Sindh, Pakistan

**Keywords:** Sindhi, NLP, Computational linguistics, Morphology, Lexicon, Dataset

## Abstract

Sindhi Unicode-8 based linguistics data set is multi-class and multi-featured data set. It is developed to solve the natural languages processing (NLP) and linguistics problems of Sindhi language. The data set presents information on grammatical and morphological structure of Sindhi language text as well as sentiment polarity of Sindhi lexicons. Therefore, data set may be used for information retrieving, machine translation, lexicon analysis, language modeling analysis, grammatical and morphological analysis, Semantic and sentiment analysis.

**Table 1** Specifications of Data set

**Table:** 

Subject area	*Natural Languages Processing*
More specific subject area	*Tagging, syntactic, Sentiment and Morphology Analysis of Sindhi Text*
Type of data	*Textual*
How data was acquired	*Corpus is taken from Sindhi newspapers, blogs and social media sites like*
	• http://sindhsalamat.com/ • http://awamiawaz.com/ • https://thefocus.wordpress.com/ • http://www.thekawish.com/beta/
	*The corpus is processed for NLP operations such as sentiment and morphological analysis, UPOS and SPOS tagging, lemma and stemming identification.*
Data format	*Data is in csv format*
Experimental factors	*Tagging, syntactic parsing, sentiment analysis, morphological analysis, lemmatization, stemming and lexicon analysis.*
Experimental features	*Unigram based analysis, token analysis, Tagging with UPOS and SPOS, Sentiment classification and morphological classification and analysis, Lemma and stemming identification*
Data source location	*Karachi, Sindh, Pakistan*
Data accessibility	*Data set may be downloaded from*http://www.sindhinlp.com/*and github*

**Value of the data**

• Data set is developed on basis of acquired results of Sindhi online natural languages processing (NLP) tool for parsing, tagging, morphological and sentiment analysis, stemming and lemmatization of Sindhi text.
• Data set is valuable to comprehend the grammatical, sentimental, syntactic and morphological structure of Sindhi text.
• Dataset is significant source for machine learning and NLP analysis for information retrieving, language modeling, machine translations, sentiment analysis and computational linguistics operations.

## 1 Data

**Fig. 1:**
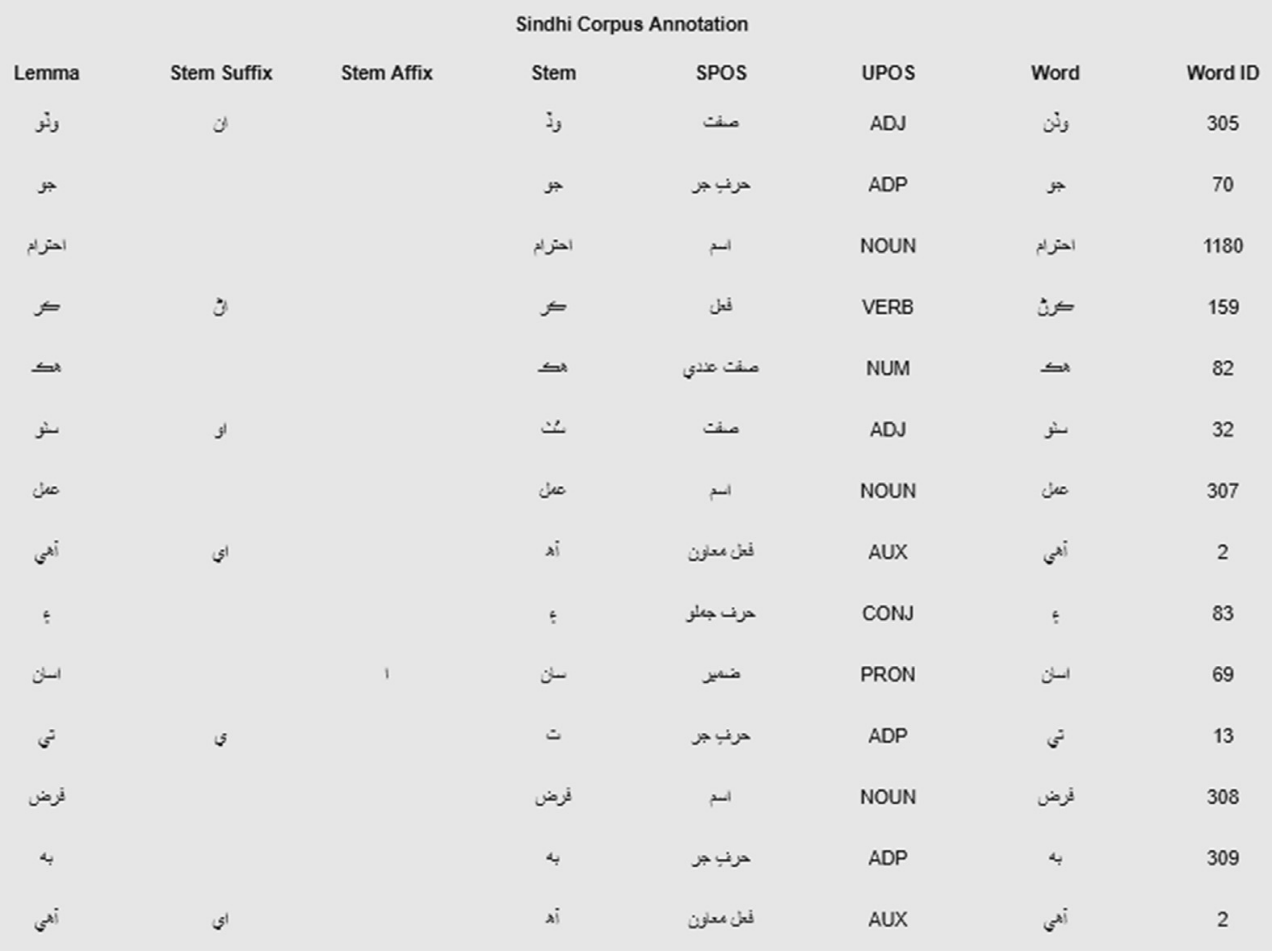
Annotation process for Sindhi corpus.

More research work has been done on English language [1] thus, lot of NLP resources are available for English language, which are not suitable for other languages such as Sindhi language. Right hand written languages are also important for NLP applications, machine and deep learning processes. Sindhi language is right hand written language and using Arabic-Persian writing style [2]. A good number of websites, blogs and social media pages are available on world wide web (www), thus, there is very good number of data available for computational linguistics, NLP, machine translations, information retrieving and machine learning processing. Polarity, UPOS annotation, SPOS annotation, Lemma and Stemming process for Sindhi text. Sindhi NLP tools are used to annotate Sindhi corpus for various purposes like tagging, sentiment analysis, lemma and stemming identification and etc. Fig. 1 shows annotation process for Sindhi text  (Waddan jo ahtaraam karann hik sutho amal aahay aen asaan te farz be aahay).

**Fig. 2:**
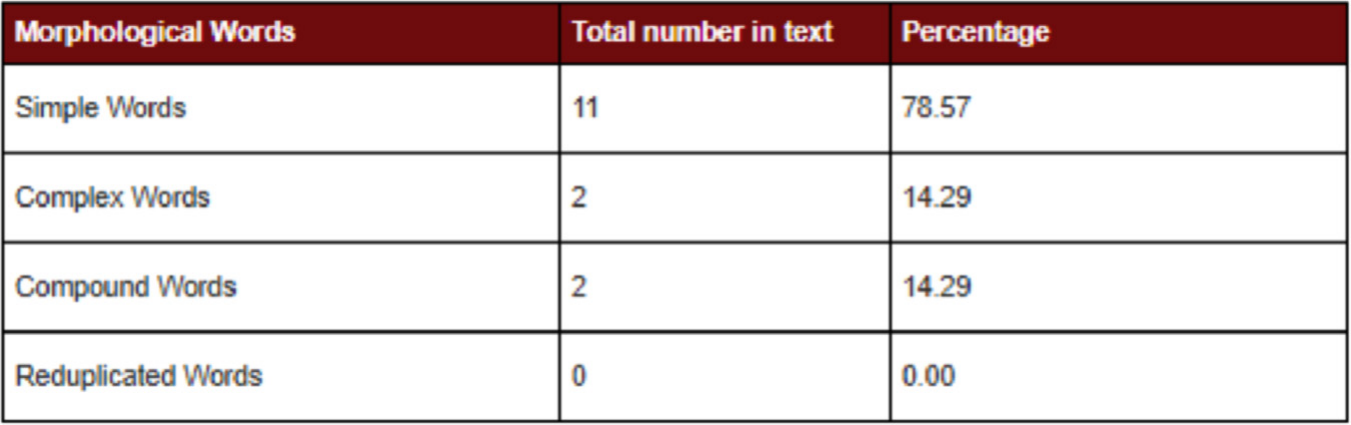
Morphological analysis of Sindhi corpus.

Fig. 2 shows the morphological analysis of Sindhi text   (Waddan jo ahtaraam karann hik sutho amal aahay aen asaan te farz be aahay).

**Fig. 3:**
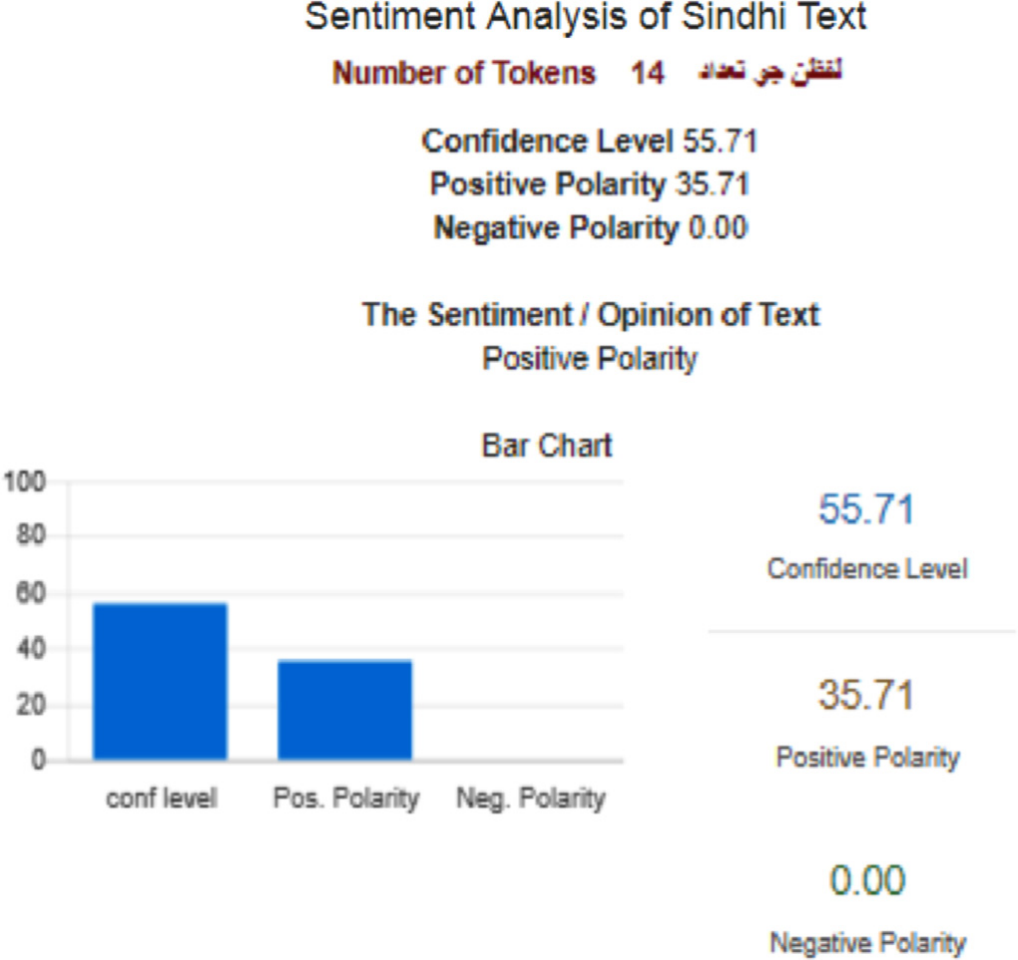
Sentiment analysis of Sindhi corpus.

Fig. 3 shows the sentiment analysis [3]of Sindhi text   (Waddan jo ahtaraam karann hik sutho amal aahay aen asaan te farz be aahay)

The dataset is consisted of 19 attributes and 6841 records. Target classes of dataset are categorical therefore, it may be good for supervised analysis. Table 1 shows the statistical analysis of Class attributes of dataset.

**Table 1:** Statistics of Sindhi annotated dataset.

*Statistics*	*UPOS*	*SPOS*	*Gender*	*Number*	*Polarity*	*Morphology*	*Lemma*	*Diacritic*	*Infinitive*
*Count*	6617	6528	6841	6841	6841	6841	6841	6841	6841
*Mean*	5.52	5.54	0.33	0.98	2.27	1.23	0.711	0.02	0.02
*Std*	4.36	4.37	0.65	0.45	1.08	0.63	0.45	0.15	0.15

## 2 Experimental design, materials and methods

Sindhi corpus documents are processed for annotation and sentiment analysis in Sindhi NLP tool separately. The results of annotation and sentiment analysis are accumulated to develop dataset. Unigram model is used to find probability of each lexicon in corpus. Dataset is processed for normalization and statistical analysis. There is no missing value found in the dataset. Brief introduction of attributes is given below:

1. **UPOS**: Universal Part of speech tag set [4], [5] is used to annotate the Sindhi tokens. UPOS is class attribute, which is consisted of 18 categories. Sindhi tokens are tagged properly with UPOS tag set. For example, Sindhi sentence  (Mango is good fruit.) may be tagged with UPOS and Sindhi part of speech (SPOS) as shown in Table 2.

**Table 2:** UPOS tagging to Sindhi text.

**Fig. 4:**
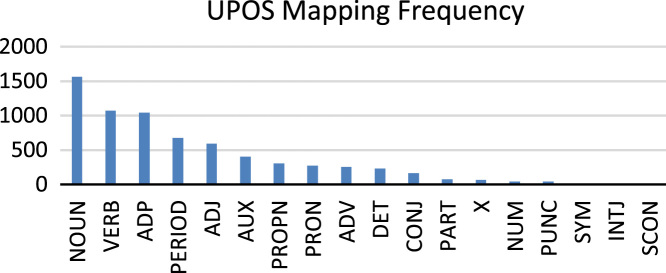
Frequency of UPOS tagged to Sindhi tokens.

The frequency of UPOS tags is dissimilar from each other in the dataset, which shows the divergence of Sindhi lexicons. Fig. 1 shows the frequency of UPOS tag set, annotated to Sindhi tokens. Fig. 4 presents the high number of Nouns and low number of Subordinating conjunction.

2. **SPOS**: Sindhi Part of Speech (SPOS) tag set is indigenous Sindhi language tag set. SPOS is class attribute of the dataset and consisted of 17 categories. There is little difference between UPOS tag PART and SPOS tag Adverb and Preposition. PART POS annotates Sindhi negation and possessive lexicons, whereas, SPOS adverb POS annotates Sindhi negation lexicons and Preposition POS annotates possessive markers available in Sindhi language, therefore, Sindhi adverb and preposition are used in place of PART POS of UPOS tag set. Sindhi treebank is novel contribution to NLP because it is not used properly for the purpose of computational linguistics operations.

The frequency of SPOS tags is different than the frequency of UPOS tags because of difference of UPOS tag PART and SPOS tag Adverb. Table 3 shows the annotation process of SPOS to Sindhi text.

**Table 3:** SPOS tagging to Sindhi text.

**Fig. 5:**
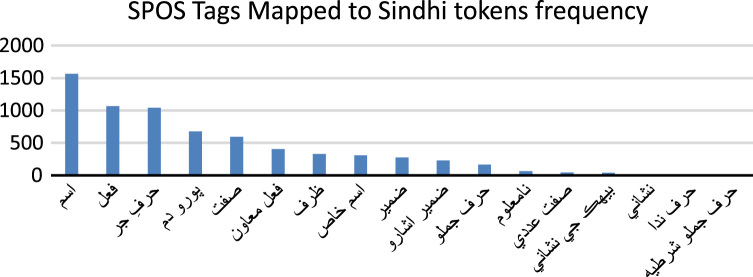
Frequency of SPOS tagged to Sindhi tokens.

Fig. 5 shows the frequency of Sindhi POS tag set which annotated to Sindhi text document   (Mango is good fruit.). There is high number of Nouns  and low number of frequency of Subordination conjunction  found in the dataset. The difference of frequency is obvious in UPOS Adverb and SPOS Adverb .

3. **Gender**: According to Sindhi Grammar [6], [7], there are two types of Gender. One is masculine called  **(Jins Muzkar)** in Sindhi and second is feminine called  (**Jins Moans)** in Sindhi. Noun, adjective and diacritic change the position of gender from masculine to feminine and vice versa. This attribute is class attribute and presents the lexicons with its proper gender. Table 4 shows the examples of Sindhi lexicons and their gender.

**Table 4:** Mapping of genders to Sindhi lexicons.

**Fig. 6:**
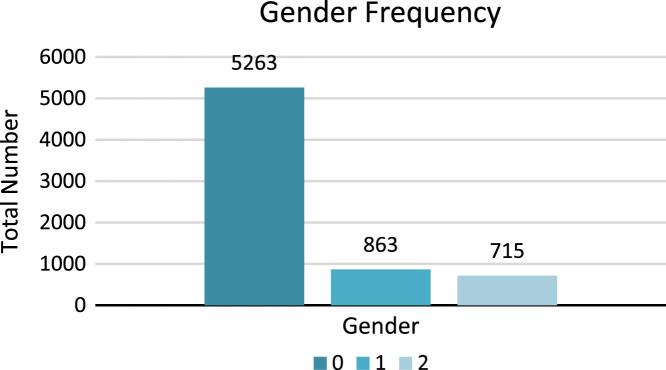
Frequency of gender types.

This dataset shows Masculine gender with digit 1, feminine gender with digit 2 and digit 0 shows no gender, which is used for periods, punctuations and symbols. Fig. 6 shows the number of frequencies of gender types.

4. **Number:** Number is of two types in Sindhi Grammar [6], [7]. One is singular  and second is plural . This feature of Sindhi dataset shows the singular or plural status of lexicon. Diacritics and extensions of words such as  make the plural number of nouns, pronouns and adjectives. Table 5 shows the status of singular and plural numbers of Sindhi text.

**Table 5:** Mapping of singular and plural numbers to Sindhi lexicons.

**Fig. 7:**
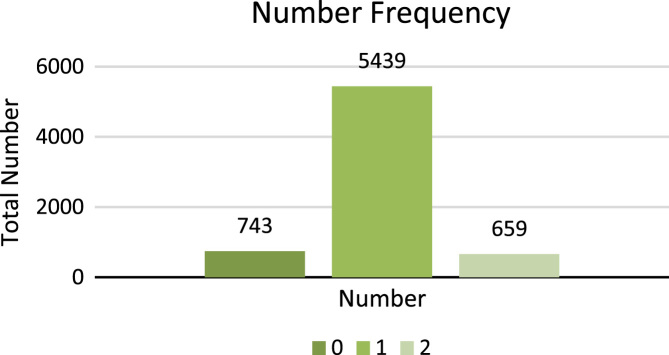
Total frequency of number types.

Singular number of noun, pronoun and adjective is shown with digit 1 and plural number of noun, pronoun and adjective is shown with digit 2 whereas, o shows no number, which is used for periods, punctuations and symbols. Fig. 7 shows the total number of frequencies of singular and plural number types.

**Fig. 8:**
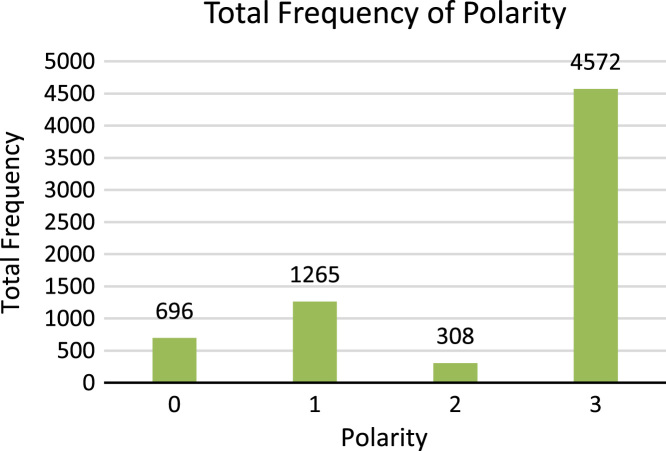
Total frequency of number types.

**Fig. 9:**
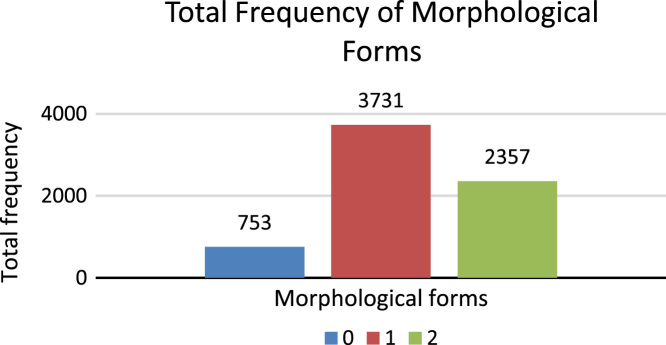
Total frequency of morphology forms.

5. **Polarity**: Polarity is class feature of the dataset, which is comprised of three categories: Positive, Negative and Neutral. All these types of polarity show the sentiment of lexicons. For example Sindhi lexicon  (Sutho) shows positive polarity,  (Kharaab) shows negative polarity and  (Theek) shows neutral polarity. For example, Sindhi sentence  **(Anb mitho mevo aahay)** shows positive polarity because adjective  (Mitho) shows positive sentiment. Digit 1 shows positive polarity, digit 2 shows negative polarity, digit 3 shows neutral polarity whereas, 0 shows no polarity which is used for periods, punctuations and symbols. Fig. 8 shows the total number of frequencies of Polarity types.

6. **Morphology:** This feature of dataset presents the morphological form of Sindhi lexicon. The attribute shows both forms of morphology, which are free form and bound form. For example, Sindhi lexicon  (trust) is a free form and  (ever) is a bound form. Bound form may be called secondary form which is divided into three categories [7], Complex, Compound and Reduplicated. Free form is shown with digit 1 and bound or secondary form is shows with digit 2 whereas, digit 0 is used for punctuations, symbols and periods. Fig. 9 shows the total number of frequencies of Morphology forms.

a. **Complex words** : Addition of affix or suffix to free form lexicon makes it the complex word of bound form. For example, Sindhi lexicon  (jjaann) may be complex word by adding affix **اَ** (aa) which changes the word  Digit 1 shows status of lexicon as complex word and digit 0 shows no complex word.
b. **Compound words** **:** It is combination of two free forms, which shows single meaning. For example, Sindhi compound word  (House Owner) is combination of two free forms,  (ghar) means House and  (Dhanni) which means owner. This attribute shows the feature of Sindhi lexicon that either it is compound or not. Compound lexicons show with digit 1 and non-compound lexicons are shows with digit 0.
c. **Reduplicated words** **:** There is little number of reduplicated words in this data set. Reduplicated words are basically compound words but the structure and presentation of these words are changed from compound words. For example, Sindhi words   and etc. are reduplicated words of bound form. Reduplicated words are the feature of Sindhi text, which make Sindhi text complex to process for NLP and computational linguistics operations. This dataset presents reduplicated words with digit 1 and no reduplicated words with digit 0.

**Fig. 10:**
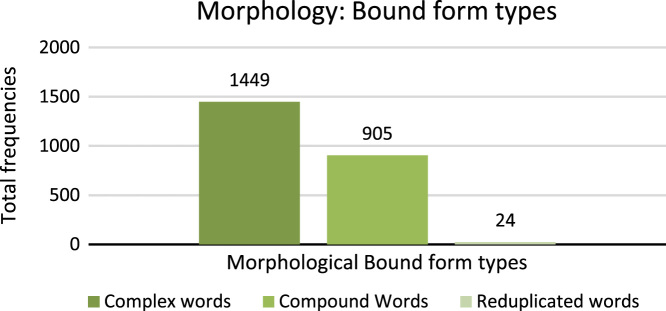
Total frequency of morphology bound form types.

Fig. 10 shows the total number of frequencies of Morphology bound form types.

**Fig. 11:**
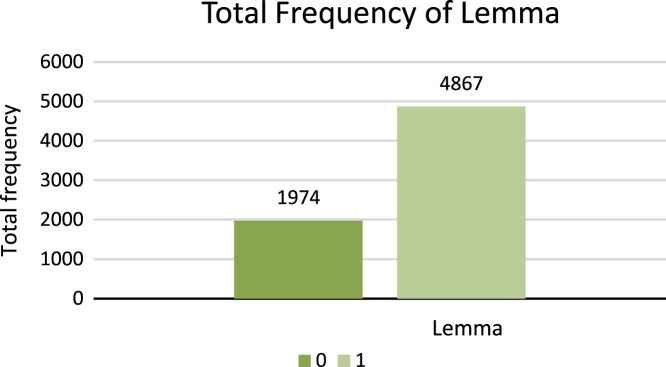
Total frequency of lemma.

**Fig. 12:**
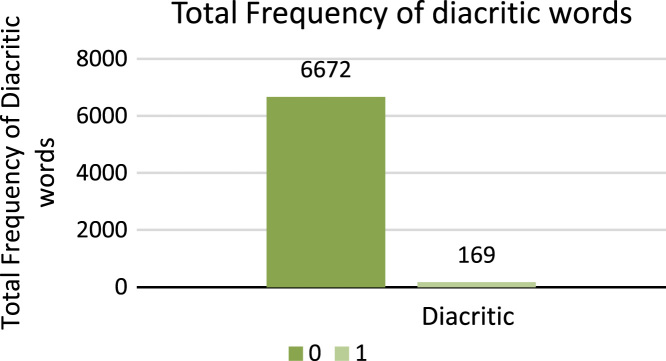
Total frequency of diacritic words.

7. **Lemmatization**: Lemmatization is process of identifying the original lexicon by reducing affix or suffix which holds grammatical and morphological structure whereas, stemming reduce the inflections, diacritics, affixes and suffixes of lexicon and derive root word. For example, Sindhi word  (To come) is complex word, therefore, the lemma of this word is  (come). Word  (Achu) shows complete meaning with proper grammar and morphological structure. Digit 1 shows lexicon as lemma and digit 0 shows no lemma. Fig. 11 shows the total number of frequencies of Lemma in the dataset.

8. **Diacritic**: Diacritic changes meaning of lexicon by attaching glyph to word or letter. For example, Sindhi lexicon  (was) is changed to another Sindhi lexicon  (he) by attaching glyph **ُ** to Sindhi letter  (ha). The diacritic changes the meaning and grammatical structure of Sindhi lexicon  (was). The first Sindhi lexicon  (was) is verb which shows action happened in past and second Sindhi lexicon  (he) is determiner. This attribute of dataset shows the diacritic feature of Sindhi lexicon with digit 1 and shows no diacritic feature with digit 0. Fig. 12 shows the total number of frequencies of diacritic words.

9. **Infinitive**: Sindhi linguists give importance to infinitive verbs called in Sindhi  (Massdar). Sindhi infinitive verbs are generated by attaching suffix to stemming or lemma words. Table 6 shows the example of Sindhi infinite verbs.

**Table 6:** Example infinitive verbs of Sindhi text.

**Fig. 13:**
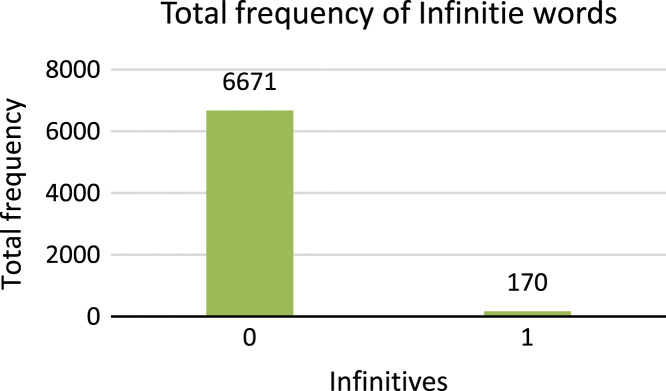
Total frequency of diacritic words.

Fig. 13 shows the total number of frequencies of infinitive words available in Sindhi annotated dataset. 0 shows non-infinitive words and 1 shows infinitive words

10. **Sindhi Token:** This attribute is significant attribute of the dataset because all features and target classes are related to this attribute. Tokens are taken from Sindhi corpus. Sindhi tokens are of following types.
  a. Sindhi lexicons.
  b. Punctuations, Symbols and Periods.

Uni-gram probability is measured by applying statistical language model to display the impact of Sindhi tokens in the dataset.
