# Supplementary material for: Unicode-8 based linguistics data set of annotated Sindhi text
Source: Data Brief. 2018 May 22;19:1504–14. doi: 10.1016/j.dib.2018.05.062 (PMC6139473; doi:10.1016/j.dib.2018.05.062)
Supplement: Supplementary file 1 — Supplementary material [file mmc1.docx]

**Conflict of interest**

All the author confirms on no Conflict of interest.
